# The impact of primary care funding on health inequalities: an umbrella review

**DOI:** 10.1017/S146342362500012X

**Published:** 2025-02-28

**Authors:** Ian Holdroyd, Lucy McCann, Maya Berger, Rebecca Fisher, John Ford

**Affiliations:** 1 Wolfson Institute of Population Health, Queen Mary University of London, London, UK; 2 Croydon University Hospital, London, UK; 3 The Health Foundation, London, UK

**Keywords:** funding, general practice, inequalities, primary care

## Abstract

**Background:**

The funding of primary care is subject to intense debate internationally. Three main funding models predominate: capitation, pay-for-performance, and fee-for-service. A number of systematic reviews regarding the effect of primary care funding structures have been published, but not synthesized through an equity lens. Given the urgent need for evaluating funding models and addressing inequalities, a reliable, synthesized evidence base concerning the effects of funding on inequalities is imperative.

**Aims:**

This umbrella review aims to systematically evaluate all systematic reviews available on the effect of different primary care funding models in high-income countries on inequalities in funding, access, outcomes, or experience from inception until 2024.

**Methods:**

Three databases (MEDLINE, EMBASE, Cochrane) and a machine learning living evidence map were searched. Abstracts and titles were double screened, before two authors independently screened full texts, extracted data, and performed quality assessments utilizing the AMSTAR2 tool.

**Findings:**

The search identified 2480 unique articles, of which 14 were included in the final review. Only one review compared reimbursement systems; capitation systems were more equitable between ethnic groups compared to pay-for-performance in terms of primary care access, continuity, and quality. Twelve reviews reviewed the impact of the introduction of pay-for-performance models, predominantly focusing on the Quality and Outcomes Framework (QOF) in the UK. Synthesized findings suggest that QOF’s introduction coincided with reduced socioeconomic health inequalities in the UK overall, but not in Scotland. Overall, inequalities in age narrowed, but inequalities measured by sex widened. One review found evidence that targeting funding for minority groups, with poorer health, was effective. A further review found that introducing privately provided general practices in Sweden and allowing patients to choose these over public-owned options generally benefitted those with higher income and lower health needs. We identify a range of gaps in the literature, which should inform future research.

## Introduction

Inequalities in health and primary care quality are well documented internationally and are a vital issue for policymakers to tackle (Marmot, 2020, LaVeist *et al.*, 2023, Karanikolos and Kentikelenis, 2016). General practitioner (GP) surgeries in poorer areas have fewer doctors per head of population and are more likely to receive poor quality ratings (The Health Foundation, 2020a, Salant *et al.*, 2024). Patients living in poorer areas are more likely to report worse experiences of access to general practice, and lower overall satisfaction with the services they receive (The Health Foundation, 2020b). Primary care inequalities are driven by an unequal distribution of the structural determinants of primary care; namely, funding, workforce, and population need (Salant *et al.*, 2024, McLean *et al.*, 2015, Kontopantelis *et al.*, 2018).

While health inequalities can be narrowed by ambitious policies that target the unequal distribution of health’s social determinants (Barr *et al.*, 2017, Buck and Maguire, 2015, Vodden *et al.*, 2023, Holdroyd *et al.*, 2022), concurrent action must ensure that inequalities across healthcare systems are addressed. Addressing inequalities in funding is imperative given its impact on service provision, workforce, and primary care quality (Salant *et al.*, 2024, L’Esperance *et al.*, 2017, McLean *et al.*, 2015). For example, in the UK, when funding across the National Health Service was increased and weighting for socioeconomic deprivation improved, this was associated with a reduction in mortality and mortality inequalities (Barr *et al.*, 2014).

Primary care is predominantly financed by three groups of funding models (Tao *et al.*, 2016). Capitation funding pays primary care providers a set fee per capita, irrespective of the services provided. Given different care needs between patients, the set fee, or patient numbers are adjusted based on a range of factors (usually referred to as ‘weighted capitation’). These differ -between health systems, but can commonly include age, sex, morbidity, and socioeconomic status (Khezri *et al.*, 2022). Pay-for-performance schemes reimburse primary care organizations for achieving pre-specified targets of activity or clinical outcomes, such as achieving 85% vaccination coverage or cervical screening. Fee-for-service models reimburse primary care organizations per item of clinical service delivered, such as a fee for each post-natal check completed. Some have argued that a fee-for-service model is beneficial due to its simplicity and feasibility (Ikegami, 2015), whereas others suggest that pay-for-performance is a beneficial mechanism to ensure funding translates into outcomes (Roland and Olesen, 2016). Others prefer capitation because it allows better adjustment for population needs (James and Poulsen, 2016). Primary care organizations are often funded by multiple funding models; this is known as blended financing.

There are widespread efforts internationally to reduce health inequalities, and much evidence that primary care has an important role to play in this (Gkiouleka *et al.*, 2023, Roland and Everington, 2016, McLean *et al.*, 2015). Understanding how structural features of primary care – for example, how health systems arrange funding for primary care services – impact health inequalities is an important step to maximizing primary care’s ability to reduce health inequalities.

Several systematic reviews have examined the effect of different models of primary care funding, with some investigating their impact on health inequalities. However, conclusions differ widely, and reviews have not been synthesized. This paper addresses this research gap by evaluating all published reviews that examine the effect of primary care funding models on inequalities in funding, access, clinical outcomes, or experience.

## Methods

This umbrella review was conducted in accordance with the established methodology (Higgins *et al.*, 2019) and reported in line with the Preferred Reporting Items for Systematic reviews and Meta-Analyses statement (Page *et al.*, 2021). The review was preregistered on PROSPERO (CRD42024501203).

### Search strategy and selection criteria

Three electronic databases (Ovid Medline, OVID Embase, and Cochrane Reviews) were systematically searched, with no time restrictions, on the 14th of January 2024. Search terms were based on existing search terms for identifying reviews (Gkiouleka *et al.*, 2023), primary care (Gkiouleka *et al.*, 2023), and inequalities (Prady *et al.*, 2018). Search terms regarding funding were based on a range of systematic reviews, forming the most comprehensive search available. The appendix presents the search terms and full search results. Following the manual removal of duplicates, all abstracts and titles were independently screened by two researchers according to inclusion and exclusion criteria using the software Rayyan with conflicts resolved through discussion.

The inclusion criteria were:

- Systematic reviews that used a structured search and included primary studies.
- Reviews in any language.
- Reviews assessing the impact of primary care funding.
- Reviews assessing outcomes of inequalities in funding, access, experience, or clinical outcomes.
- Reviews in high-income countries as defined by the world bank.

Exclusion criteria were:

- Primary research studies.
- Reviews not investigating inequalities.
- Reviews reporting non-health inequalities.

Further papers were identified from the Health Equity Evidence Centre’s Living Evidence Maps; a machine learning resource to identify and collate studies that focus on what works to address inequalities in primary care (Health Equity Evidence Centre, 2024). Additionally, all citations of included reviews were screened.

The full text of all identified articles were reviewed by two researchers. For all reviews that met inclusion criteria, the following information was extracted: first author, year of publication, aim, number of studies, time period of analysis, population, primary care funding model(s) being investigated, health inequalities measured, and main findings. Outcomes of interest were any change or difference in inequality occurring due to a change in primary care funding or difference in primary care funding. Quality assessment was performed using the AMSTAR2 tool (Shea *et al.*, 2017). Data extraction and quality assessment were performed independently by two authors before any disagreements were resolved with discussion with a third author.

Due to the small number of reviews, a large amount of data heterogenicity, and lack of data in systematic reviews, it was inappropriate to perform meta-analysis. Instead, the review’s findings were narratively synthesized. When reviews differed in their conclusions about the effects of the same intervention, efforts were made to understand the reasons behind these differences and reach a consensus. This involved assessing several factors including whether different definitions were used to frame outcomes, the primary studies identified in each review, the weight assigned to individual studies’ findings, unique considerations specific to each review, and the overall quality of the review. Any significant findings that helped explain the differences in conclusions were incorporated into the narrative synthesis.

## Results

**Figure 1.**
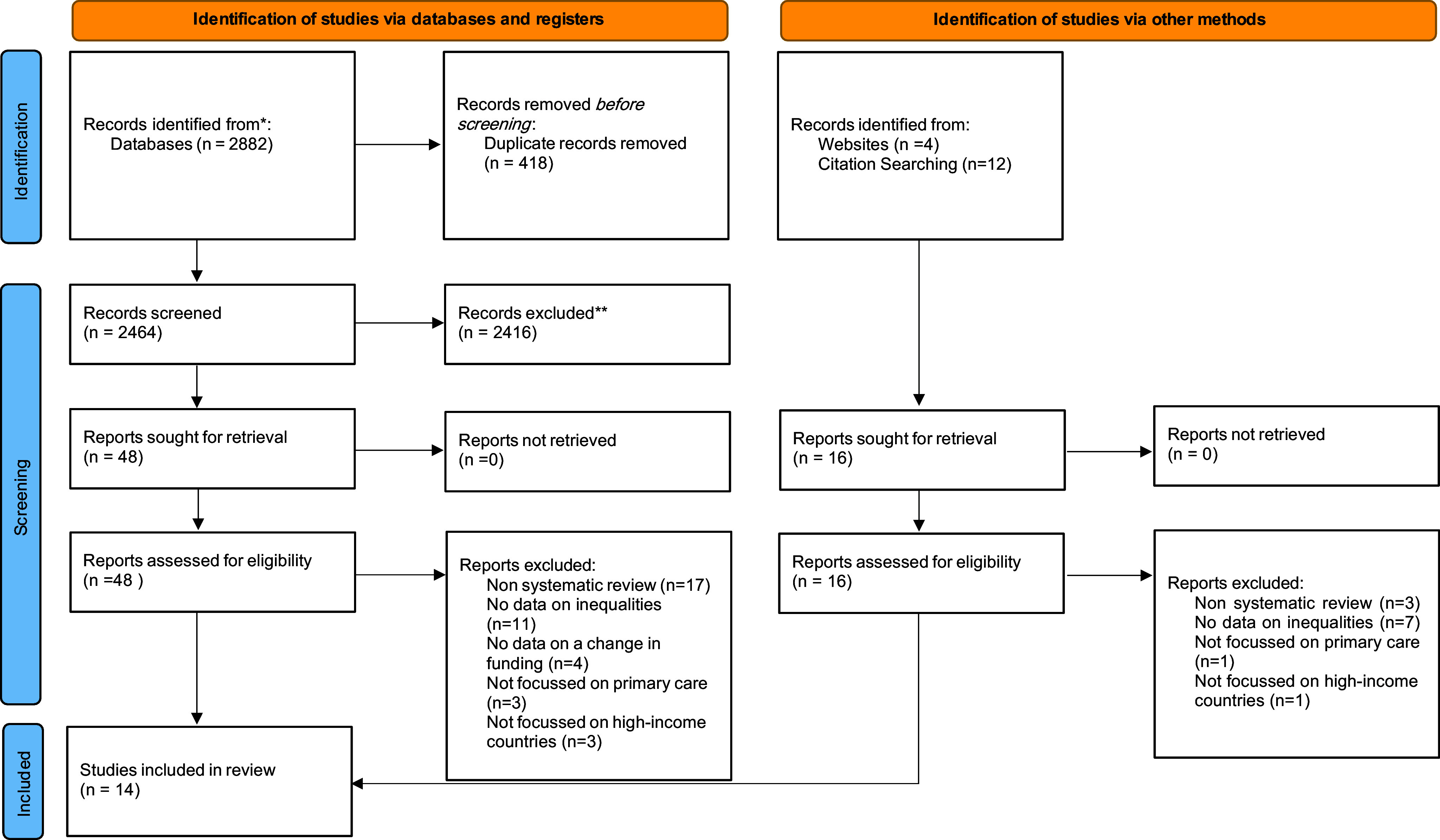
PRISMA diagram of study selection criteria.

After the removal of duplicates, the search identified 2480 unique articles. Sixty-four were reviewed in full text, and 14 were included in the final review. Figure 1 shows a flowchart of included reviews.

The 14 reviews were published between 2009 and 2021 (Table 1). Only one compared funding models, comparing capitation reimbursement to fee-for-service (Tao *et al.*, 2016). Twelve reviews investigated the effects of pay-for-performance reimbursement (Ahmed *et al.*, 2021, Alshamsan *et al.*, 2010, Boeckxstaens *et al.*, 2011, Forbes *et al.*, 2016, Gillam *et al.*, 2012, Gupta and Ayles, 2020, Lin *et al.*, 2016, Mandavia *et al.*, 2017, Steel and Willems, 2010, Tao *et al.*, 2016, Van Herck *et al.*, 2010, Annemans *et al.*, 2009). One review investigated the effects of Swedish reform to allow privately funded primary care and increased patient choice of provider (Burstrom *et al.*, 2017). One review investigated the effects of fee-for-services and the effect of targeting funding to minority groups (Gibson and Segal, 2015). Results, and quality assessments, are summarized in Table 2. Full quality assessment results can be found in the appendix.

**Table 1.** Characteristics of all included reviews

Author, year	Aim	Number of studies (study publication dates)	Population or organizational group	Change in primary care funding investigated by studies
Reviews investigating only the impact of pay-for-performance				
Boeckxstaens *et al.* (2011)	To describe changes in inequalities in healthcare following the introduction of the Quality and Outcomes Framework (QOF)	19 of 27 studies were conducted on data from England, 6 from Scottish data, and 2 studies used combined data (2004–2009)	UK residents registered with general practice	Introduction of QOF
Annemans *et al.* (2009)	To investigate the international pay-for-performance literature regarding their design, implementation, and evaluation.	104, of which 32 are concerned with equity. Of these, 27 investigated the equity impacts of the QOF (2006–2008)	Healthcare providers in general primary and/or hospital care	Effects of the QOF
Lin *et al.* (2016)	To review pay-for-performance on both the behaviour of primary care doctors and patient outcomes	44, 20 on equity (1998–2013)	15 on populations in England concerning the impact of QOF, 1 in French patients, 1 in the Taiwanese population for pay-for-performance, 2 in the USA, 1 in the Netherlands	The impact of the introduction of any pay-for-performance system
Steel and Willems (2010)	To review the effects of the QOF on healthcare processes and outcomes, including equity.	10 assessing equity. Study dates NA	English and Scottish residents registered with general practice introduction.	Introduction of QOF
Alshamsan *et al.* (2010)	To assess the impact of pay-for-performance programmes on the quality of health care in relation to age, sex, ethnicity, and socioeconomic status	22, 21 focused on changes to primary care, 20 QOF, and 1 other UK change. (2003–2008)	UK residents registered with general practice and participating in QOF	Introduction of pay-for-performance programmes
Forbes *et al.* (2016)	To review evidence that QOF maintains or increases the quality of primary medical care, and to consider potential downsides of QOF	A total of 40 studies included, 2 primary studies included evidence on the equity effects of QOF (2013, 2014)	Two repeated measure studies of General Practices before-and-after introduction of QOF	Introduction of the QOF
Mandavia *et al.* (2017)	To review financial incentives (capitation, fee-for-service, salary, and block budgets) in improving healthcare in the UK	28, 21 assessed the effects of QOF. Equity findings from 5 papers reported. (2009–2016)	Individual patient and practice-level data from the UK	Effects of the QOF
Van Herck *et al.* (2010)	Explored: how pay-for-performance schemes impact a range of outcomes including equity and how this is affected by the design of a pay-for-performance scheme	128, 28 studies investigated equity (1995–2009	63 studies were conducted in the USA, 57 in the UK, 2 in Australia, 2 in Germany, 2 in Spain, 1 in Argentina, and 1 in Italy. No information regarding the studies investigating equity specifically	Pay-for-performance schemes
Gupta and Ayles (2020)	To review pay-for-performance among primary care physicians for diabetes management and the impact of patients’ and/or physicians’ sex/gender	39, 12 studies reported sex and gender inequalities. 2 studies described the impact of the QOF (2009)	Patients from Canada (province of New Brunswick), Italy (Emilia-Romagna region), Taiwan, and the United Kingdom.	Introduction of pay-for-performance and fee-for-service programmes, 20 papers investigating QOF, 1 a US pay-for-performance programme, and 1 another UK
Gillam *et al.* (2012)	To review the impact of the QOF on the quality of primary medical care	2006–2011	English and Scottish residents registered with general practice	Introduction of QOF
Ahmed *et al.* (2021)	To review incentive schemes available in general practice in the UK and their subsequent impact and effectiveness in changing the quality of care and identify other forms of funding incentives in the literature	35, 3 findings with respect to inequalities. Study dates unavailable	UK residents registered with general practice	Pay-for-performance schemes
Reviews investigating other changes in primary care funding				
Tao *et al.* (2016)	To assess the effect of different reimbursement systems on socioeconomic and ethnic inequalities in access, utilization, and quality of primary care	22 (2004–2013)	Patients in the UK, England, Scotland, Canada, and the USA	Capitation models compared to fee-for-service Introduction of pay-for-performance schemes (QOF)
Gibson and Segal (2015)	To review the impact of primary care initiatives on health outcomes of Indigenous people with type 2 diabetes in Australia, NZ, Canada, and the USA	13, 3 investigated the effect of changes in funding. (2001–2010)	Indigenous people in the USA, Australia, and NZ All three were cohort studies	Three studies increased total primary care funds and facilitated the transfer of funds to local health organizations or Indigenous groups for the purchase and delivery of health services. One operated a pay-for-services model whereby general practitioners (GPs) or practice nurses were reimbursed for completed diabetes care plans delivered in accordance with guidelines. The second transferred funds to Indigenous health organizations or tribal groups. The third financed local primary care providers to employ practice nurses and provide patients with a wellness plan and health consultations
Burstrom *et al.* (2017)	To review the effect of the Swedish Primary Health Care 2010 Choice Reform on equity	Six articles and nine reports (2008–2016)	Sweden residents	Introduction of choice reform which allowed citizens to choose their primary healthcare service provider and allowed private providers of primary health care to establish practices

**Table 2.** Review findings, sorted by aim and quality assessment result

Author, year, title	Main findings and conclusions	Quality assessment
Reviews investigating only the impact of pay-for-performance		
Boeckxstaens *et al.* (2011)	● For **socioeconomic** inequalities, there was initially a small widening in inequity after the introduction of the Quality and Outcomes Framework (QOF); however, this narrowed in the years after. This narrowing was due to initially poorer initial performance for practices in deprived areas, rather than their location in a deprived area per se. For some indicators, large differences remained. ● With respect to **ethnicity** there was no clear pattern as to the patient groups benefitted by the introduction of QOF. A range of measures for each ethnic group showed examples where outcomes were improved relative to other groups, and examples where outcomes did not improve or became worse relative to other groups. No ethnic group was clearly benefitted or negatively impacted by the change. ● The introduction of QOF widened inequalities between **sexes**. For a range of outcomes for coronary heart disease (CHD), cerebrovascular disease (CVD), and diabetes, a pre-QOF difference favouring males persisted, and for other measures, a pro-male inequity was noted following QOF’s introduction. ● QOF narrowed inequalities in inequality measured by **age**. Pre-QOF, younger patients generally had improved outcomes in a range of indicators of CHD, CVD, and diabetes. These improved more for older, compared to younger patients.	Moderate
Annemans *et al.* (2009)	● For **socioeconomic** inequalities, the gap in overall quality of care (measured by total QOF score) between more and less deprived areas narrowed in the years following the QOF’s introduction. This was due to poorer initial performance, rather than being located in a deprived area per se. In the years following the introduction of the QOF, there was a decrease in the difference in rates of recording blood pressure between practices in the most and least deprived areas. ● Mixed results were found regarding the relationship between exception reporting and **socioeconomic** measures of deprivation. Exception reporting is when a GP can exclude a patient from their list when calculating their outcome quality. The most recent included study found that patient characteristics explained only a limited percentage of variance. ● Before the QOF, there were worse outcomes for medical processes, and patient outcomes for older patients for CVD, CHD, and diabetes. Following the introduction of QOF, the total number of indicators with inequity by **age** was decreased. There was less evidence regarding whether QOF decreased inequalities when measured by sex, socioeconomic status, and ethnicity.	Moderate
Lin *et al.* (2016)	● Results were not narratively synthesized. Instead, the effects of pay-for-performance on inequalities were summarized. ● For **socioeconomic** inequalities, six studies reported inequalities widened, three reported no effect, and two reported inequalities narrowed. ● For **ethnicity**, six studies reported a widening of inequality, two no effect, and two reported inequalities narrowed. ● For **sex** inequalities, five reported that inequalities widened and one reported inequalities narrowed. ● For **age**, nine studies reported that inequalities widened and three reported inequalities narrowed. ● For **comorbidity**, four studies reported inequalities widened, one no change, and four reported that inequalities narrowed. ● For practice size, five studies reported inequalities widened, 1 no effect, and three reported inequalities narrowed.	Moderate
Steel and Willems (2010)	● Reported that for **socioeconomic** inequalities, results were different when investigating for England and Scotland. In both, practices in more deprived areas had lower quality indicator scores in the first year post-QOF. In England, these gaps present in the first year after QOFs introduction narrowed and almost disappeared in the years following. Large gaps remained for some indicators. In contrast, in Scotland, there was a lower increase in quality indicators in the most deprived areas, resulting in a widening of inequity. ● Changes in inequalities by **ethnicity** were varied, with no consistent pattern. ● Gaps in a range of achievement scores by **sex** persisted or widened. ● For inequalities of **age**, gaps in care for CHD, diabetes, and CVD improved after QOFs introduction due to greater improvement in older patients.	Low
Alshamsan *et al.* (2010)	● Reported that the introduction of QOF was associated with reductions in **socioeconomic** inequalities in chronic disease management between affluent and deprived areas. Initially, there were lower QOF scores in more deprived areas, but this difference attenuated in the second and third years of QOF. ● Argues that inequalities in ‘medical processes’ in **age** and **sex** and a range of measures of **ethnicity** persisted, specifically that women, older patients, and those from some minority ethnic groups continued to receive lower quality of care after the introduction of QOF. ● A more limited pay-for-performance scheme introduced in 1999 provided financial incentives for reaching cervical cancer screening targets. This was associated with widening **socioeconomic** inequalities between deprived and affluent areas, though these were largely attenuated at five years follow-up.	Low
Forbes *et al.* (2016)	● The two primary studies did not find a clear effect of the QOF introduction on **socioeconomic** inequalities of emergency admission rates by income deprivation, nor on medical processes and outcomes for type 2 diabetics when measured by **age**, **gender**, **socioeconomic** position, or **comorbidities**. ● One study found that individual patients were more likely to be exception reported, if they were older, less affluent, and had multiple morbidities. These patients were more likely to die in the following year.	Low
Mandavia *et al.* (2017)	● Did not narratively synthesize findings from the five studies. ● Reported that one negative impact of the QOF was widened inequalities by **ethnicity** and **age**.	Low
Van Herck *et al.* (2010)	● The specific aims, methods, and findings of included studies are not provided nor discussed. ● Authors reported that across 28 studies, in general, pay-for-performance did not have negative effects on inequalities by **socioeconomic status**, **ethnicity**, **age**, or **comorbidities**. ● Reported widened inequalities by **sex** – there was less improvement for females as compared to male patients.	Low
Gupta and Ayles (2020)	● Described two studies relating to QOF. The first reported that in the following QOF’s introduction. The first showed a narrowing of **socioeconomic** inequalities. The second showed a widening of inequalities by the number of **comorbidities** a patient has.	Low
Gillam *et al.* (2012)	● Not all studies were narratively synthesized: Only 4 of 25 studies concerning equity were included in narrative synthesis to support statements. ● Reported a narrowing of **socioeconomic** inequalities and inequalities of **age**. Reported that disparities between **sex** for CVD and diabetes persisted or widened and that variations in care by **ethnicity** have been narrowed. ● Suggested that the ability to exclude (exception report) some patients from the QOF population, who are likely harder to reach, may result in a limitation on its impact on health inequalities.	Low
Ahmed *et al.* (2021)	● Identified two studies that investigated the effects of QOF on health inequalities. ● Briefly report findings from a systematic review included in this study, reporting a narrowing of **socioeconomic** health inequalities as a result of QOF. ● Reported one study where patients with more **comorbidities** benefited from QOF while those without were negatively impacted. ● One study assessed pay-for-performance in a socioeconomically deprived area. Results suggested that GPs in less affluent areas were motivated by financial incentives, while in more affluent areas they were motivated more by patient benefit.	Critically low
Reviews investigating other changes in primary care funding		
Tao *et al.* (2016)	● Six studies comparing capitation to fee-for-service were discussed. ● Overall, healthcare plans that were capitation-based had reduced inequalities of **ethnicity** in access, continuity (measured as having a ‘usual source of care’), and a measure of quality of care (ambulatory care sensitive admissions). One study reported that in a capitation plan had was greater inequity in minority ethnic groups reporting lower patient satisfaction than white people. The outcomes were communication skills and how thorough the physician was. Of 12 outcomes, 2 were significant: (1) worse explanations given to Hispanic patients when interviewed in English and (2) worse thoroughness in Hispanic patients when interviewed in English. There were no significant effects for the other ten outcomes, including Hispanic patients being interviewed in Spanish. One study found no difference by **ethnicity** or **socioeconomic** inequalities in mortality between patients of a fee-for-service and capitation care plans. ● Six studies investigated the effects of the introduction of QOF. Studies found that this had little or no effect on socioeconomic and inequity of **ethnicity** in the management of CVD, COPD, diabetes, and preventive services.	Moderate
Gibson and Segal (2015)	● Three studies investigated the effect of making more money available to local groups and health services within Indigenous communities to increase their ability for health purchasing. Two achieved a reduction in HbA1c in these groups, reducing inequity of **ethnicity**. ● The first involved the transfer of funds to Indigenous health organizations or tribal groups, and the second financed local primary care providers to employ practice nurses and provide patients with a wellness plan and health consultations. The third study, whereby primary care providers were paid on a fee-for-service basis for each diabetic care plan that was made, failed to show improvement.	Moderate
Burstrom *et al.* (2017)	● Regarding **socioeconomic** inequalities, some evidence suggested that new practices were more likely to be founded in areas with more affluent populations. Some contrasting evidence was also found. The number of visits of patients to primary care increased more for high-income earners. Patient satisfaction was higher in more affluent areas. ● Regarding inequalities of **age**: some evidence found that practices were more likely to be found in areas with fewer older adults. ● Regarding **comorbidity**, the number of primary care visits increased more for those with lower health needs, widening inequality. The Primare Care reforms resulted in barriers to integrated care for those with greater health needs.	Low

‘Medical processes’: a process done at the GP surgery, for example, recording smoking status, recording blood pressure; ‘prescribing’: patients being offered a drug they are eligible for; ‘outcomes’, for example, Hb1Ac, blood pressure; ‘quality of care’: total QOF points; ‘COPD’: combined obstructive pulmonary disease.

### Results comparing different funding models

One moderate quality review of six studies explored inequalities in access, continuity and quality of care, and patient satisfaction of patients in capitation-based plans compared to fee-for-service (Tao *et al.*, 2016). Two US studies and one Canadian study found that capitation-based plans had reduced inequalities in access to care between ethnic groups. Both US studies also found reduced inequalities in continuity of care and ambulatory care sensitive condition admissions between ethnic groups in capitation-based plans compared to fee-for-service. In contrast, one study assessing the quality of physician’s communication skills found reduced ethnic inequalities in two of twelve measures of patient satisfaction in a fee-for-service care plan compared to capitation. One study found no effect on ethnic or educational inequalities in mortality.

### Pay-for-performance: the quality and outcomes framework

The 12 reviews that examined the equity impact of pay-for-performance reimbursement focused primarily on the effect of the introduction of the Quality and Outcomes Framework (QOF) in the UK. All reviews included primary studies investigating the QOF, and seven concentrated on the QOF alone (Boeckxstaens *et al.*, 2011, Forbes *et al.*, 2016, Gillam *et al.*, 2012, Mandavia *et al.*, 2017, Steel and Willems, 2010, Tao *et al.*, 2016, Annemans *et al.*, 2009). This scheme was introduced in 2004 with general practices receiving payment for achieving a range of standards. These standards include (1) medical processes (e.g. taking blood pressure readings, offering smoking cessation advice), (2) prescribing relevant medication, and (3) improving patient outcomes (e.g. blood pressure, Hba1c).

The reviews included studies that used a range of before-and-after, serial cross-sectional, and cross-sectional analyses and examined various outcomes such as clinical processes, prescribing, and outcomes. Before-and-after analysis was facilitated by comparing pre- and post-QOF quality of care. Pre-QOF quality of care was measured by practice performance on a range of non-incentivized quality indicators (including medical processes, prescribing, and clinical outcomes) similar or identical to QOF indicators. The most commonly assessed conditions were cardiovascular disease, diabetes, coronary heart disease, hypertension, asthma, combined obstructive pulmonary disease, stroke, and preventative health. The review’s conclusions varied significantly.

Regarding socioeconomic inequalities, two reviews reported that pay-for-performance schemes reduced inequalities (Ahmed *et al.*, 2021, Gillam *et al.*, 2012), whereas another concluded that it had no effect (Tao *et al.*, 2016). Other papers, with generally higher quality assessment scores, and a larger number of included papers, offered more nuanced findings (Alshamsan *et al.*, 2010, Annemans *et al.*, 2009, Boeckxstaens *et al.*, 2011, Steel and Willems, 2010). Primary studies typically investigated socioeconomic inequalities by comparing patients living in more or less affluent areas, as measured by deprivation scores. A minority of studies focused on the occupation of patients. Synthesized results of these reviews suggested that in the first year following the introduction of QOF, there were temporarily reduced scores in less affluent areas, representing a temporary widening of inequality. This difference attenuated in the second and third years of QOF, indicating that inequalities narrowed (Alshamsan *et al.*, 2010, Annemans *et al.*, 2009, Boeckxstaens *et al.*). Only one review investigated differences across the UK, finding that this narrowing effect was not seen in Scotland, where there was less improvement in quality indicators in the most deprived areas, causing a widening of inequality. (Steel and Willems, 2010). For some indicators, large inequalities still remain (Boeckxstaens *et al.*, 2011, Steel and Willems, 2010).

Five reviews synthesized findings on inequalities of age. One review reported that QOF widened inequalities, in favour of older patients who had better quality of care (Mandavia *et al.*, 2017). However, this review, scored as low quality, only identified two studies, with outcomes that were better in older patients at the time of the QOF. The remaining four reviews, which had a wider literature base, reported that before QOF, older patients generally had worse quality of care compared to younger patients when measured by a range of medical processes, prescribing, and patient outcomes (Alshamsan *et al.*, 2010, Boeckxstaens *et al.*, 2011, Steel and Willems, 2010, Annemans *et al.*, 2009). One reported that inequalities persisted in a range of medical processes following the introduction of QOF (Alshamsan *et al.*, 2010), while three reported a narrowing of inequalities (Boeckxstaens *et al.*, 2011, Steel and Willems, 2010, Annemans *et al.*, 2009). The former review, reporting the persistence of inequalities, did indeed report findings of primary studies that generally showed a narrowing of inequalities however chose to assess QOF’s success based on whether inequalities disappeared, rather than narrowed. Therefore overall, QOF’s introduction coincided with reduced inequalities in age, favouring older patients who had a worse quality of care before the QOF.

Five reviews reported synthesized findings regarding inequalities between sexes. At the time of QOF’s implementation, men generally had higher quality of care measured by clinical process, prescription, and clinical outcomes (Alshamsan *et al.*, 2010, Boeckxstaens *et al.*, 2011, Steel and Willems, 2010). One study found limited evidence of improved inequalities, (Annemans *et al.*, 2009) and the other four reviews concluded that QOF’s implementation corresponded with a widening of inequalities (Alshamsan *et al.*, 2010, Boeckxstaens *et al.*, 2011, Gillam *et al.*, 2012, Steel and Willems, 2010). Pro-male pre-QOF differences persisted or widened. For some inequalities that did not have a difference between sexes, a pro-male difference emerged in the years following QOF’s implementation.

Five reviews synthesized findings regarding ethnicity. Overall, there was no clear pattern of changes in inequalities following QOF’s implementation (Alshamsan *et al.*, 2010, Boeckxstaens *et al.*, 2011, Steel and Willems, 2010, Tao *et al.*, 2016, Annemans *et al.*, 2009).

Multiple reviews noted the existence of gaming of the QOF; several highlighted exception reporting as a limitation. Exception reporting permits practices to exclude certain patients from target calculations based on patient compliance, disease status, medication suitability, availability of services, or patients being new to the practice. This is designed to factor in patient autonomy and prevent practices from losing potential funding due to factors outside of their control. However, findings indicated that it is possible for GPs to game this process and that there are unintended consequences: patients who are older, less affluent, and multi-morbid are slightly more likely to be exception reported, and these patients are more likely to die in the following year (Forbes *et al.*, 2016, Gillam *et al.*, 2012, Annemans *et al.*, 2009).

### Reviews investigating other pay-for-performance schemes

Five reviews also investigated the effects of other pay-for-performance schemes (Ahmed *et al.*, 2021, Alshamsan *et al.*, 2010, Gupta and Ayles, 2020, Lin *et al.*, 2016, Van Herck *et al.*, 2010). Findings were limited by a number of factors, most notably a lack of primary studies that were not focusing on the QOF. Additionally, review quality limited findings: one review’s quality was scored as critically low, and three were scored as low. In the two reviews which had a notable number of studies not concerning the QOF, conclusions were limited by a lack of qualitative description of the type of schemes and their outcomes, given the wide heterogenicity of these factors (Lin *et al.*, 2016, Van Herck *et al.*, 2010).

### Reviews investigating other changes to primary care funding

One review explored changes in inequity occurring after Swedish health reform in 2008–10 where privately funded primary healthcare practices could be founded and citizens could choose amongst primary care providers (Burstrom *et al.*, 2017). Overall, patterns suggested increased inequality due to patients with lower health needs benefiting more from these changes. Private practices were most likely to be found in areas with populations with lower health needs. Additionally, while access increased for all groups it did so more for those with lower health needs. Reforms acted as a barrier to integrated care for those with greater health needs.

One review found evidence that increasing funding specifically targeted at minority groups, with poorer health, was effective in narrowing inequalities (Gibson and Segal, 2015). Three primary studies identified Indigenous groups in the USA, Australia, and New Zealand, who had worse type 2 diabetes clinical outcomes compared to the national average. Of the two studies that were successful in improving health outcomes (measured by improved HbA1c), both involved the unconditional transfer of financial resources to facilitate improved care. The third, which utilized a fee-for-service model, where providers were paid per diabetic care plan, failed to show a narrowing in inequalities.

## Discussion

### What this research means

Capitation models were more equitable than fee-for-service plans for patient access, continuity, and quality of care. This however is not to say that all capitation systems are necessarily more equitable. Capitation models differ globally, and research did not identify what factors make capitation models more equitable. For example, in the UK, the capitation formula is over 20 years old. Many argue that it is outdated and does not sufficiently account for clinical needs (Roland and Everington, 2016, Kontopantelis *et al.*, 2018). It has been suggested that – by failing to weigh the additional health needs of people who live in poorer areas – this formula compounds funding inequalities: areas with higher deprivation scores receive less funding relative to their population’s higher health needs (Kontopantelis *et al.*, 2018, McLean *et al.*, 2015, Roland and Everington, 2016). Recent evidence underscores this point: adjusting capitation funding in England by deprivation data would improve funding equity (Holdroyd *et al.*, 2024). The use of capitation models alone, therefore, is not sufficient to reduce health inequalities- individual capitation models must be well designed to ensure that such an outcome occurs.

Sufficient assessment of pay-for-performance schemes was limited to that of the QOF. Overall, conclusions varied widely – likely due to variation in the number of studies identified in reviews (2–32 concerning equity) and varying levels of review quality (critically low to moderate). In the first year of QOF’s introduction, there appeared to be an initial widening of inequality when comparing the quality of primary care (measured by QOF indicators) in practices in more and less deprived areas. Differences in performance by levels of deprivation narrowed and attenuated over the following years in England, but not in Scotland. Inequalities in age narrowed, while inequalities by sex persisted or widened. No evidence indicated that these changes were due to the QOF itself, rather than other wider societal factors. Reviews and primary studies focused on the early impact of QOF’s introduction and not its ongoing effect on health equity.

It was notable that socioeconomic inequalities narrowed in England, but not in Scotland following QOF’s introduction. At this time, there was a National Health Inequalities Strategy in England, which was conducted between 1999 and 2010. This was successful in narrowing socioeconomic health inequalities (Barr *et al.*, 2017, Buck and Maguire, 2015, Vodden *et al.*, 2023, Holdroyd *et al.*, 2022). Given that this strategy only applied to England, but not devolved nations, this may explain why narrowed socioeconomic inequalities were only seen in England. Such evidence indicates the ability of the central government to reduce health inequalities and strengthens calls for future strategies internationally.

Evidence that directing funding to specific disadvantaged communities can improve their health and has implications for a range of countries internationally for whom large health inequalities exist between Indigenous populations and the rest of the country. This evidence suggested unconditional transfers (where money is given regardless of the outcome), rather than fee-for-service funding, were most effective.

In Sweden, allowing privately operated general practices to open without oversight or equity-promoting financial incentives resulted in some measures of increased inequality.

### Strengths and limitations

This umbrella review is the first to collate and synthesize all reviews of the effect of different primary care funding models on health inequalities. This review’s main strength is in its robust and preregistered methodology, with an extensive search strategy followed by a robust screening, data extraction, and quality assessment process completed by two authors. We included a wide range of peer-reviewed articles and grey literature. Such an approach allows the synthesis of a wide literature base and consensus to be formed on the debated effects of a range of policy interventions, most notably, QOF. This approach allows not just consensus on these issues but identifies a range of issues currently underexplored in the literature.

The quality assessment identified several limitations within the reviews. AMSTAR criteria highlighted key collective weaknesses, most notably the lack of a republished methodology, insufficient explanation of study designs for inclusion, failure to consider funding sources, and inadequate accounting for the risk of bias. The overall quality of the evidence base was significantly weakened, as many reviews relied on primary studies that produced correlational rather than causal results. For example, the effects of QOF cannot be seen in isolation given that until 2010, the government was simultaneously committed to reducing socioeconomic health inequalities, with a range of ambitious policy interventions that improved social determinants of health, health behaviours, and other inequalities in funding (Barr *et al.*, 2017, Vodden *et al.*, 2023, Barr *et al.*, 2014, Holdroyd *et al.*, 2022). Such conclusions are common when evaluating the effect of national policy. Research from more federal systems, with regional differences in policy, would strengthen the literature base.

### Comparison to previous research

One previous umbrella review aimed to explore the effects of pay-for-performance in health care, with one of six aims regarding inequalities (Eijkenaar *et al.*, 2013). This review concluded that pay-for-performance schemes result generally in reduced rather than increased socioeconomic health inequalities, but not inequalities by age, sex, or ethnicity. These findings were drawn from a limited literature base, with only four reviews identified. While we find similar evidence regarding the narrowing of socioeconomic health inequalities following QOF’s implementation, we identify many limitations of the literature that prevent such generalized statements from being made regarding the impact of pay-for-performance. Additionally, our review’s findings indicate that the period following QOF’s implementation saw a widening of inequalities by sex and a narrowing of inequalities by age. A second review also included systematic reviews; however, it identified only one of the reviews included in this umbrella review (Forbes *et al.*, 2016). This review had limited conclusions due to a lack of evidence concerning inequalities being identified. This review builds on these findings by identifying a larger literature base, exploring a wider range of changes in funding, and exploring reasons for differing results.

### Implications for policy and research

Current research focuses predominantly on QOF; more research is needed on a wider range of funding models. For example, only one review examined the inequity effects associated with capitation models, despite their widespread adoption globally (Khezri *et al.*, 2022). Despite targeted search terms, no research was identified for salaried payment models. This less commonly used model pays primary care providers per hour worked. It has less relevance in socialized healthcare systems as an individual is more likely to act within an organization that receives other forms of payment. Further research could address this by reviewing evidence of its effect on health inequalities. Additionally, research could investigate the impact of blended funding models.

Many factors could improve the strength of research. Addressing the reliance on cross-sectional, observational evidence necessitates a shift towards quasi-causal methodologies, such as the difference-in-difference approach (Cha and Escarce, 2022). Current research lacks granularity, exploring the overarching effects of funding changes without delving into their underlying components. Key areas of interest include the effects of different amounts of funding, the target recipients of funding (ranging from large institutions to individual practitioners), and the utilization of financial incentives versus penalties. Furthermore, reviews are predominantly dated, and an updated review would ensure that new primary studies are identified. Only one review compared different funding models. Further analysis understanding the differential effect of these funding models, especially on socioeconomic health inequalities, would further deepen understanding of their respective impacts.

Results regarding exception reporting widening inequality were concerning. To address these issues, exception reporting mechanisms should be updated to prevent GPs from measuring their performance against only patients who likely received better quality of care. Examples include reducing the time that patients can be excluded after joining the list or being less permissive of GPs not engaging with patients – currently, three letters through the post is sufficient for excepting.

Some evidence suggests that following a change in the funding model that requires action by primary care organizations, there may be an immediate short-term widening of inequality. This was seen in the first year after QOF’s introduction and also after a pay-for-performance scheme introduced in the UK in 1999 to incentivize cervical cancer screening targets (Alshamsan *et al.*, 2010, Boeckxstaens *et al.*, 2011, Steel and Willems, 2010). In both cases, these inequalities narrowed over the following years. Reasons suggested for this vary. It may be that in more affluent areas practices can better respond to change due to more capacity as a result of lower clinical need. Alternatively, the inverse equity hypothesis was suggested, whereby affluent groups in society preferentially benefit from any societal interventions, and less affluent groups only benefit once the maximum benefit has been extracted (Victora *et al.*, 2000). Regardless of the causal reason, the potential for short-term increases in inequality should be considered when interventions occur, and contingencies for this should be made.

### Conclusion

This umbrella review highlights evidence indicating the effect of primary care funding on inequalities in healthcare access, experience, and clinical outcomes. Some evidence indicated the pro-equity benefits of capitation models over fee-for-service models. A range of previously contested evidence concerning the effect of the QOF was synthesized, finding that overall, socioeconomic inequalities in the quality of primary care narrowed over the years following implementation. A stronger and more diverse literature base would more easily allow policy decisions to be driven by strong evidence.

## Supporting information

Holdroyd et al. supplementary material 1 Holdroyd et al. supplementary material

Holdroyd et al. supplementary material 2 Holdroyd et al. supplementary material

## Supplementary material

The supplementary material for this article can be found at https://doi.org/10.1017/S146342362500012X.
