# The impact of primary care funding on health inequalities: an umbrella review – CORRIGENDUM

**DOI:** 10.1017/S1463423625000283

**Published:** 2025-03-26

**Authors:** Ian Holdroyd, Lucy McCann, Maya Berger, Rebecca Fisher, John Ford

This article was originally published with an incorrect author affiliation.

The affiliation of John Ford was given as Croydon University Hospital, London, UK. The correct affiliation is Wolfson Institute of Population Health, Queen Mary University of London, London, UK.

The original article has been updated.

The authors and publisher apologise for this error.

## References

[ref1] Holdroyd I , McCann L , Berger M , Fisher R , Ford J. The impact of primary care funding on health inequalities: an umbrella review. Primary Health Care Research & Development. 2025;26:e24. doi: 10.1017/S146342362500012X PMC11883797 40017117

