# Supplementary material for: The impact of primary care funding on health inequalities: an umbrella review
Source: Prim Health Care Res Dev. 2025 Feb 28;26:e24. doi: 10.1017/S146342362500012X (PMC11883797; doi:10.1017/S146342362500012X)
Supplement: Holdroyd et al. supplementary material 1 — Holdroyd et al. supplementary material [file S146342362500012Xsup001.docx]

| Question | Ahmed et al. (2021) | Alshamsan et al. (2010) | Annemans et al. (2009) | Boeckxstaens et al. (2011) | Burstrom et al. (2017) | Forbes et al. (2016) | Gibson and Segal (2015) | Gillam et al. (2012) | Gupta and Ayles (2020) | Lin et al. (2016) | Mandavia et al. (2017) | Steel and Willems (2010) | Tao et al. (2016) | Van Herck et al. (2010) |  |
| --- | --- | --- | --- | --- | --- | --- | --- | --- | --- | --- | --- | --- | --- | --- | --- |
| 1 | N | N | Y | N | N | Y | Y | N | Y | Y | N | N | Y | Y |  |
| 2 | N | N | N | N | N | N | N | N | Y | N | N | N | N | N |  |
| 3 | N | N | N | N | N | N | N | N | N | N | N | N | N | N |  |
| 4 | PY | PY | PY | PY | N | Y | PY | PY | PY | PY | PY | N | PY | PY |  |
| 5 | Y | NA | Y | Y | N | Y | Y | N | PY | Y | Y | N | Y | Y |  |
| 6 | N | Y | Y | Y | N | Y | N | Y | Y | N | Y | N | N | N |  |
| 7 | N | N | PY | N | N | N | N | N | Y | N | N | N | N | PY |  |
| 8 | N | NA | Y | N | N | Y | Y | NA | N | PY | Y | NA | Y | N |  |
| 9 | N | Y | PY | N | N | N | PY | PY | PY | PY | PY | PY | PY | PY |  |
| 10 | N | N | N | N | N | N | N | N | N | N | N | N | N | N |  |
| 11 | NA | NA | NA | NA | NA | NA | NA | NA | NA | NA | NA | NA | NA | NA |  |
| 12 | NA | NA | NA | NA | NA | NA | NA | NA | NA | NA | NA | NA | NA | NA |  |
| 13 | Y | N | N | N | N | N | N | N | N | N | N | N | N | N |  |
| 14 | N | Y | Y | Y | N | N | Y | N | N | N | N | Y | Y | N |  |
| 15 | NA | NA | Y | N | NA | NA | NA | NA | NA | NA | NA | NA | NA | NA |  |
| 16 | Y | N | Y | N | Y | N | N | N | Y | Y | Y | N | Y | Y |  |
| Overall Confidence | Critically Low | Low | Moderate | Moderate | Low | Low | Moderate | Low | Low | Moderate | Moderate | Low | Moderate | Low |  |

Supplementary Table 1- Full Results of Quality Assessments completed with AMSTAR2 tool. N: No; PY: Partial Yes; Y: Yes; NA: Not Applicable
