# Supplementary material for: The impact of primary care funding on health inequalities: an umbrella review
Source: Prim Health Care Res Dev. 2025 Feb 28;26:e24. doi: 10.1017/S146342362500012X (PMC11883797; doi:10.1017/S146342362500012X)
Supplement: Holdroyd et al. supplementary material 2 — Holdroyd et al. supplementary material [file S146342362500012Xsup002.docx]

Supplementary Material 2: Full Literature Search Results

Medline:

Ovid MEDLINE(R) and Epub Ahead of Print, In-Process, In-Data-Review & Other Non-Indexed Citations, Daily and Versions <1946 to January 12, 2024>

1 Meta-Analysis as Topic/ or meta analy$.tw. or metaanaly$.tw. or Meta-Analysis/ or (systematic adj (review$1 or overview$1)).tw. or exp Review Literature as Topic/ or cochrane.ab. or embase.ab. or (psychlit or psyclit).ab. or (psychinfo or psycinfo).ab. or (cinahl or cinhal).ab. or science citation index.ab. or bids.ab. or cancerlit.ab. or reference list$.ab. or bibliograph$.ab. or hand-search$.ab. or relevant journals.ab. or manual search$.ab. or ((selection criteria or data extraction).ab. and Review/) or (review or systematic review).pt. or review*.ti. 3755140

2 (primary care or primary health care or primary healthcare or (general practi* or family practi* or family medicine or GP)).mp. or exp primary health care/ or exp general practice/ or exp family practice/ or exp physicians, family/ or exp general practitioners/ 459469

3 exp "Health Expenditures"/ or exp "Reimbursement Mechanisms"/ or exp "Physician Incentive Plans"/ or (reimburs* or remunerat* or fee* or capitat* or salar* or "Outcomes Framework*" or QOF or pay* or fund* or "global sum" or global-sum or "Carr Hill" or Carr-Hill or Incentiv*).mp. 1603785

4 Cultural Deprivation/ or Acculturation/ or Culture/ or Cross-Cultural Comparison/ or Cultural Characteristics/ or Cultural Diversity/ or Language/ or "Transients and Migrants"/ or exp "Emigrants and Immigrants"/ or Minority groups/ or Minority health/ or Prejudice/ or Racism/ or Xenophobia/ or Social Discrimination/ or exp Race Relations/ or exp Ethnic Groups/ or exp Continental Population Groups/ or Refugees/ or minorit*.mp. or migration background.mp. or racial.mp. or racism.mp. or ethnology.mp. or race.mp. or ethnic*.mp. or non?English.mp. or language other than.mp. or latino*.mp. or latina*.mp. or hispanic*.mp. or whites.mp. or caucasian*.mp. or non?white.mp. or Torres Strait Islander.mp. or aboriginal.mp. or native american.mp. or inuit.mp. or eskimo.mp. or first nation*.mp. or indigenous.mp. or english as a second language.mp. or foreign language.mp. 850721

5 Residence Characteristics/ or Environment design/ or exp Marital status/ or neighbo?rhood*.mp. or residential environment*.mp. or rural*.mp. or inner?city.mp. or housing instability.mp. or housing insecurity.mp. or housing strain.mp. or housing security.mp. or mortgage problems.mp. or foreclosure.mp. or eviction*.mp. or housing loss.mp. or home repossession*.mp. or home ownership.mp. or (repossess* adj3 hous*).mp. or (repossess* adj3 propert*).mp. or mortgage delinquency.mp. or mortgage arrears.mp. or mortgage debt*.mp. or overcrowding.mp. or (living adj1 (outside or inside or near* or adjacent)).mp. or (household adj2 size).mp. or (marital status or marriage status).mp. or (widow* or cohabit* or divorce* or single parent* or live* alone).mp. 368267

6 Occupations/ or Unemployment/ or occupations.mp. or unemployment.mp. 67672

7 exp Gender Identity/ or Women's Health/ or gender differences.mp. or (sex disparit* or sex difference?).mp. or gender identity.mp. or sex role.mp. or wom#n* role?.mp. or m#n* role?.mp. or gender* role?.mp. or servicewomen.mp. or Sex factors/ 399822

8 exp Educational status/ or Education/ or Schooling.mp. or educational status.mp. or (education* adj2 level?).mp. or ((higher or better or worse or less) adj educated).mp. or ((higher or better or worse or less) adj level? of education).mp. 156995

9 Religion/ or religi*.mp. 74182

10 Social determinants of Health/ or Psychosocial Deprivation/ or Sociological Factors/ or Working Poor/ or Hierarchy, Social/ or disparit*.mp. or inequalit*.mp. or inequit*.mp. or equity.mp. or deprivation.mp. or gini.mp. or concentration index.mp. or Socioeconomic Factors/ or Social Welfare/ or exp Social Class/ or exp Poverty/ or Income/ or Social class*.mp. or social determinants.mp. or social status.mp. or social position.mp. or social background.mp. or social circumstance*.mp. or socio-economic.mp. or socioeconomic.mp. or sociodemographic.mp. or socio-demographic.mp. or SES.mp. or disadvantaged.mp. or impoverished.mp. or poverty.mp. or economic level.mp. or assets index.mp. or income*.mp. 860499

11 Social Stigma/ or social capital/ or Social Control, Informal/ or exp Social Support/ or exp Social Environment/ or Trust/ or Social conditions/ or Social isolation/ or Social marginalization/ or Anomie/ or social participation/ or social exclusion.mp. or (social adj (capital or cohes* or organis* or organiz*)).mp. or (community adj3 (cohes* or participa*)).mp. or ((neighbourhood or neighborhood) adj cohes*).mp. or social relationships.mp. or social network*.mp. or collective efficacy.mp. or civil society.mp. or informal social control.mp. or neighbo*rhood disorder.mp. or social disorgani?ation.mp. or anomie.mp. or social support.mp. or social participation.mp. or trust.mp. or emotional support.mp. or psychosocial support.mp. or community capital.mp. or neighbo*rhood cohesion.mp. or social influence.mp. or (soci*context* or soci*-context*).mp. 350299

12 Health Status Disparities/ or Health Services Accessibility/ or Health Equity/ or health*care disparit*.mp. or health care disparit*.mp. or health status disparit*.mp. or health disparit*.mp. or health inequalit*.mp. or health inequit*.mp. or medically underserved.mp. 155757

13 (potential determinants or significant correlates of or (independent correlates or independent association*) or variables associated with or determinants of or factors associated with or identif* determinants or (more likely or less likely or just as likely) or risk factors for or (significantly related to or significant predictor) or (also adj2 associated with) or (at increased risk or at decreased risk) or association* between or (positively associated or negatively associated) or differed by or (were high* amongst or were low* amongst) or (inverse relationship with or inversely associated with or inversely related to) or reverse association or differentially affects or evidence of a link between or (significantly adj3 likelihood of) or protective factors for or (differ* adj2 according to) or (inverse adj2 gradient) or (positive adj2 gradient) or (negative adj2 gradient) or (trends were adj3 across) or (related to adj3 variable*) or (differences were adj3 explained by) or (significant among or no# significant among)).mp. 2671370

14 4 or 5 or 6 or 7 or 8 or 9 or 10 or 11 or 12 or 13 4506577

15 1 and 2 and 3 and 14 1990

Embase:

Embase <1974 to 2024 January 12>

1 exp Meta Analysis/ or ((meta adj analy$) or metaanalys$).tw. or (systematic adj (review$1 or overview$1)).tw. or cancerlit.ab. or cochrane.ab. or embase.ab. or (psychlit or psyclit).ab. or (psychinfo or psycinfo).ab. or (cinahl or cinhal).ab. or science citation index.ab. or bids.ab. or reference lists.ab. or bibliograph$.ab. or hand-search$.ab. or manual search$.ab. or relevant journals.ab. or ((data extraction or selection criteria).ab. and review.pt.) 713430

2 (primary care or primary health care or primary healthcare or (general practi* or family practi* or family medicine or GP)).ti,ab. or exp *primary health care/ or exp *primary medical care/ or exp *general practice/ or exp *general practitioner/ 425869

3 exp "Health Expenditures"/ or exp "Reimbursement Mechanisms"/ or exp "Physician Incentive Plans"/ or (reimburs* or remunerat* or fee* or capitat* or salar* or "Outcomes Framework*" or QOF or pay* or fund* or "global sum" or global-sum or "Carr Hill" or Carr-Hill or Incentiv*).mp. 2388236

4 demography/ or environmental planning/ or marriage/ or divorce/ or cohabitation/ or widow/ or exp "single (marital status)"/ or neighbo?rhood*.mp. or residential environment*.mp. or rural*.mp. or inner?city.mp. or housing instability.mp. or housing insecurity.mp. or housing strain.mp. or housing security.mp. or mortgage problems.mp. or foreclosure.mp. or eviction*.mp. or housing loss.mp. or home repossession*.mp. or home ownership.mp. or (repossess* adj3 hous*).mp. or (repossess* adj3 propert*).mp. or mortgage delinquency.mp. or mortgage arrears.mp. or mortgage debt*.mp. or overcrowding.mp. or (living adj1 (outside or inside or near* or adjacent)).mp. or (household adj2 size).mp. or (marital status or marriage status).mp. or (widow* or cohabit* or divorce* or single parent* or live* alone).mp. 756522

5 exp cultural deprivation/ or cultural factor/ or cultural anthropology/ or cultural diversity/ or exp migrant/ or minority group/ or minority health/ or prejudice/ or exp social discrimination/ or exp race relation/ or exp ethnic group/ or exp ancestry group/ or exp refugee/ or minorit*.mp. or migration background.mp. or racial.mp. or racism.mp. or ethnology.mp. or race.mp. or ethnic*.mp. or non?English.mp. or language other than.mp. or latino*.mp. or latina*.mp. or hispanic*.mp. or whites.mp. or caucasian*.mp. or non?white.mp. or Torres Strait Islander.mp. or aboriginal.mp. or native american.mp. or inuit.mp. or eskimo.mp. or first nation*.mp. or indigenous.mp. or english as a second language.mp. or foreign language.mp. 1247276

6 exp employment status/ or job characteristics/ or occupations.mp. or unemployment.mp. 74313

7 exp gender identity/ or women's health/ or sex difference/ or (sex disparit* or sex difference?).mp. or gender identity.mp. or sex role.mp. or wom#n* role?.mp. or m#n* role?.mp. or gender* role?.mp. or servicewomen.mp. 527233

8 exp educational status/ or schooling.mp. or educational status.mp. or (education* adj2 level?).mp. or ((higher or better or worse or less) adj educated).mp. or ((higher or better or worse or less) adj level? of education).mp. 222503

9 religion/ or religi*.mp. 104795

10 "social determinants of health"/ or social aspect/ or working poor/ or exp social hierarchy/ or socioeconomics/ or disparit*.mp. or inequalit*.mp. or inequit*.mp. or equity.mp. or deprivation.mp. or gini.mp. or concentration index.mp. or social welfare/ or social class/ or poverty/ or social status/ or social background/ or social class*.mp. or social determinants.mp. or social status.mp. or social position.mp. 818547

11 (social background or social circumstance* or socio-economic or socioeconomic or sociodemographic or socio-demographic or SES or disadvantaged or impoverished or poverty or economic level or assets index or income*).mp. 643902

12 exp social isolation/ or social capital/ or social stigma/ or social support/ or social environment/ or trust/ or exp social exclusion/ or anomie/ or social participation/ or social exclusion.mp. or (social adj (capital or cohes* or organis* or organiz*)).mp. or (community adj3 (cohes* or participa*)).mp. or ((neighbourhood or neighborhood) adj cohes*).mp. or social relationships.mp. or social network*.mp. or collective efficacy.mp. or civil society.mp. or informal social control.mp. or neighbo*rhood disorder.mp. or ocial disorgani?ation.mp. or anomie.mp. or social support.mp. or social participation.mp. or trust.mp. or emotional support.mp. or psychosocial support.mp. or community capital.mp. or neighbo*rhood cohesion.mp. or social influence.mp. or (soci*context* or soci*-context*).mp. 409734

13 health disparity/ or health equity/ or health care access/ or health*care disparit*.mp. or health care disparit*.mp. or health status disparit*.mp. or health disparit*.mp. or health inequalit*.mp. or health inequit*.mp. or medically underserved.mp. 175370

14 (potential determinants or significant correlates of or (independent correlates or independent association*) or variables associated with or determinants of or factors associated with or identif* determinants or (more likely or less likely or just as likely) or risk factors for or (significantly related to or significant predictor) or (also adj2 associated with) or (at increased risk or at decreased risk)).mp. 1990967

15 (association* between or (positively associated or negatively associated) or differed by or (were high* amongst or were low* amongst) or (inverse relationship with or inversely associated with or inversely related to) or reverse association or differentially affects or evidence of a link between or (significantly adj3 likelihood of) or protective factors for or (differ* adj2 according to) or (inverse adj2 gradient) or (positive adj2 gradient) or (negative adj2 gradient) or (trends were adj3 across) or (related to adj3 variable*) or (differences were adj3 explained by) or (significant among or no# significant among)).mp. 1265447

16 4 or 5 or 6 or 7 or 8 or 9 or 10 or 11 or 12 or 13 or 14 or 15 5624160

17 1 and 2 and 3 and 16 787

Cochraine:

Date Run: 14/01/2024 13:49:09

Comment:

ID Search Hits

#1 [mh "Health Expenditures"] OR [mh "Reimbursement Mechanisms"] OR [mh "Physician Incentive Plans"] OR (reimburs*:ti,ab,kw OR remunerat*:ti,ab,kw OR fee*:ti,ab,kw OR capitat*:ti,ab,kw OR salar*:ti,ab,kw OR ("Outcomes" NEXT Framework*):ti,ab,kw OR QOF:ti,ab,kw OR pay*:ti,ab,kw OR fund*:ti,ab,kw OR "global sum":ti,ab,kw OR global-sum:ti,ab,kw OR "Carr Hill":ti,ab,kw OR Carr-Hill:ti,ab,kw OR Incentiv*:ti,ab,kw) 129818

#2 ("primary care":ti,ab,kw OR "primary health care":ti,ab,kw OR "primary healthcare":ti,ab,kw OR (("general" NEXT practi*):ti,ab,kw OR ("family" NEXT practi*):ti,ab,kw OR "family medicine":ti,ab,kw OR GP:ti,ab,kw)) OR [mh "primary health care"] OR [mh "general practice"] OR [mh "family practice"] OR [mh "physicians, family"] OR [mh "general practitioners"] 43495

#3 [mh ^"Residence Characteristics"] OR [mh ^"Environment design"] OR [mh "Marital status"] OR

neighbo?rhood*:ti,ab,kw OR ("residential" NEXT environment*):ti,ab,kw OR rural*:ti,ab,kw OR inner?city:ti,ab,kw OR "housing instability":ti,ab,kw OR "housing insecurity":ti,ab,kw OR "housing strain":ti,ab,kw OR "housing security":ti,ab,kw OR "mortgage problems":ti,ab,kw OR foreclosure:ti,ab,kw OR eviction*:ti,ab,kw OR "housing loss":ti,ab,kw OR ("home" NEXT repossession*):ti,ab,kw OR "home ownership":ti,ab,kw OR (repossess*:ti,ab,kw NEAR/3 hous*:ti,ab,kw) OR (repossess*:ti,ab,kw NEAR/3 propert*:ti,ab,kw) OR "mortgage delinquency":ti,ab,kw OR "mortgage arrears":ti,ab,kw OR ("mortgage" NEXT debt*):ti,ab,kw OR overcrowding:ti,ab,kw OR (living:ti,ab,kw NEAR/1 (outside:ti,ab,kw OR inside:ti,ab,kw OR near*:ti,ab,kw OR adjacent:ti,ab,kw)) OR (household:ti,ab,kw NEAR/2 size:ti,ab,kw) OR ("marital status":ti,ab,kw OR "marriage status":ti,ab,kw) OR (widow*:ti,ab,kw OR cohabit*:ti,ab,kw OR divorce*:ti,ab,kw OR ("single" NEXT parent*):ti,ab,kw OR (live* NEXT "alone"):ti,ab,kw) 19414

#4 [mh ^"Cultural Deprivation"] OR [mh ^Acculturation] OR [mh ^Culture] OR [mh ^"Cross-Cultural Comparison"] OR [mh ^"Cultural Characteristics"] OR [mh ^"Cultural Diversity"] OR [mh ^Language] OR [mh ^"Transients and Migrants"] OR [mh "Emigrants and Immigrants"] OR [mh ^"Minority groups"] OR [mh ^"Minority health"] OR [mh ^Prejudice] OR [mh ^Racism] OR [mh ^Xenophobia] OR [mh ^"Social Discrimination"] OR [mh "Race Relations"] OR [mh "Ethnic Groups"] OR [mh "Continental Population Groups"] OR [mh ^Refugees] OR minorit*:ti,ab,kw OR "migration background":ti,ab,kw OR racial:ti,ab,kw OR racism:ti,ab,kw OR ethnology:ti,ab,kw OR race:ti,ab,kw OR ethnic*:ti,ab,kw OR non?English:ti,ab,kw OR "language other than":ti,ab,kw OR latino*:ti,ab,kw OR latina*:ti,ab,kw OR hispanic*:ti,ab,kw OR whites:ti,ab,kw OR caucasian*:ti,ab,kw OR non?white:ti,ab,kw OR "Torres Strait Islander":ti,ab,kw OR aboriginal:ti,ab,kw OR "native american":ti,ab,kw OR inuit:ti,ab,kw OR eskimo:ti,ab,kw OR ("first" NEXT nation*):ti,ab,kw OR indigenous:ti,ab,kw OR "english as a second language":ti,ab,kw OR "foreign language":ti,ab,kw 48747

#5 [mh ^Occupations] OR [mh ^Unemployment] OR occupations:ti,ab,kw OR unemployment:ti,ab,kw 1797

#6 [mh "Gender Identity"] OR [mh ^"Women's Health"] OR "gender differences":ti,ab,kw OR (("sex" NEXT disparit*):ti,ab,kw

OR ("sex" NEXT difference?):ti,ab,kw) OR "gender identity":ti,ab,kw OR "sex role":ti,ab,kw OR (wom?n* NEXT role?):ti,ab,kw OR (m?n* NEXT role?):ti,ab,kw OR (gender* NEXT role?):ti,ab,kw OR servicewomen:ti,ab,kw OR [mh ^"Sex factors"] 15219

#7 [mh "Educational status"] OR [mh ^Education] OR Schooling:ti,ab,kw OR "educational status":ti,ab,kw OR (education*:ti,ab,kw NEAR/2 level?:ti,ab,kw) OR ((higher:ti,ab,kw OR better:ti,ab,kw OR worse:ti,ab,kw OR less:ti,ab,kw) NEXT educated:ti,ab,kw) OR ((higher:ti,ab,kw OR better:ti,ab,kw OR worse:ti,ab,kw OR less:ti,ab,kw) NEXT (level? NEXT "of education"):ti,ab,kw) 11027

#8 [mh ^Religion] OR religi*:ti,ab,kw 2846

#9 [mh ^"Social determinants of Health"] OR [mh ^"Psychosocial Deprivation"] OR [mh ^"Sociological Factors"]

OR [mh ^"Working Poor"] OR [mh ^"Hierarchy, Social"] OR disparit*:ti,ab,kw OR inequalit*:ti,ab,kw OR inequit*:ti,ab,kw OR equity:ti,ab,kw OR deprivation:ti,ab,kw OR gini:ti,ab,kw OR "concentration index":ti,ab,kw OR [mh ^"Socioeconomic Factors"] OR [mh ^"Social Welfare"] OR [mh "Social Class"] OR [mh Poverty] OR [mh ^Income] OR ("Social" NEXT class*):ti,ab,kw OR "social determinants":ti,ab,kw OR "social status":ti,ab,kw OR "social position":ti,ab,kw OR "social background":ti,ab,kw OR ("social" NEXT circumstance*):ti,ab,kw OR socio-economic:ti,ab,kw OR socioeconomic:ti,ab,kw OR sociodemographic:ti,ab,kw OR socio-demographic:ti,ab,kw OR SES:ti,ab,kw OR disadvantaged:ti,ab,kw OR impoverished:ti,ab,kw OR poverty:ti,ab,kw OR "economic level":ti,ab,kw OR "assets index":ti,ab,kw OR income*:ti,ab,kw 46735

#10 [mh ^"Social Stigma"] OR [mh ^"social capital"] OR [mh ^"Social Control, Informal"] OR [mh "Social Support"] OR [mh "Social Environment"] OR [mh ^Trust] OR [mh ^"Social conditions"] OR [mh ^"Social isolation"] OR [mh ^"Social marginalization"] OR [mh ^Anomie] OR [mh ^"social participation"] OR "social exclusion":ti,ab,kw OR (social:ti,ab,kw NEXT (capital:ti,ab,kw OR cohes*:ti,ab,kw OR organis*:ti,ab,kw OR organiz*:ti,ab,kw)) OR (community:ti,ab,kw NEAR/3 (cohes*:ti,ab,kw OR participa*:ti,ab,kw)) OR ((neighbourhood:ti,ab,kw OR neighborhood:ti,ab,kw) NEXT cohes*:ti,ab,kw) OR "social relationships":ti,ab,kw OR ("social" NEXT network*):ti,ab,kw OR "collective efficacy":ti,ab,kw OR "civil society":ti,ab,kw OR "informal social control":ti,ab,kw OR (neighbo*rhood NEXT "disorder"):ti,ab,kw OR ("social" NEXT disorgani?ation):ti,ab,kw OR anomie:ti,ab,kw OR "social support":ti,ab,kw OR "social participation":ti,ab,kw OR trust:ti,ab,kw OR "emotional support":ti,ab,kw OR "psychosocial support":ti,ab,kw OR "community capital":ti,ab,kw OR (neighbo*rhood NEXT "cohesion"):ti,ab,kw OR "social influence":ti,ab,kw OR soci*context*:ti,ab,kw 25339

#11 [mh ^"Health Status Disparities"] OR [mh ^"Health Services Accessibility"] OR [mh ^"Health Equity"] OR (health*care NEXT disparit*):ti,ab,kw OR ("health care" NEXT disparit*):ti,ab,kw OR ("health status" NEXT disparit*):ti,ab,kw OR ("health" NEXT disparit*):ti,ab,kw OR ("health" NEXT inequalit*):ti,ab,kw OR ("health" NEXT inequit*):ti,ab,kw OR "medically underserved":ti,ab,kw 3567

#12 ("potential determinants":ti,ab,kw OR "significant correlates of":ti,ab,kw OR ("independent correlates":ti,ab,kw OR ("independent" NEXT association*):ti,ab,kw) OR "variables associated with":ti,ab,kw OR "determinants of":ti,ab,kw OR "factors associated with":ti,ab,kw OR (identif* NEXT "determinants"):ti,ab,kw OR ("more likely":ti,ab,kw OR "less likely":ti,ab,kw OR "just as likely":ti,ab,kw) OR "risk factors for":ti,ab,kw OR ("significantly related to":ti,ab,kw OR "significant predictor":ti,ab,kw) OR (also:ti,ab,kw NEAR/2 "associated with":ti,ab,kw) OR ("at increased risk":ti,ab,kw OR "at decreased risk":ti,ab,kw) OR (association* NEXT "between"):ti,ab,kw OR ("positively associated":ti,ab,kw OR "negatively associated":ti,ab,kw) OR "differed by":ti,ab,kw OR (("were" NEXT high* NEXT "amongst"):ti,ab,kw OR ("were" NEXT low* NEXT "amongst"):ti,ab,kw) OR ("inverse relationship with":ti,ab,kw OR "inversely associated with":ti,ab,kw OR "inversely related to":ti,ab,kw) OR "reverse association":ti,ab,kw OR "differentially affects":ti,ab,kw OR "evidence of a link between":ti,ab,kw OR (significantly:ti,ab,kw NEAR/3 "likelihood of":ti,ab,kw) OR "protective factors for":ti,ab,kw OR (differ*:ti,ab,kw NEAR/2 "according to":ti,ab,kw) OR (inverse:ti,ab,kw NEAR/2 gradient:ti,ab,kw) OR (positive:ti,ab,kw NEAR/2 gradient:ti,ab,kw) OR (negative:ti,ab,kw NEAR/2 gradient:ti,ab,kw) OR ("trends were":ti,ab,kw NEAR/3 across:ti,ab,kw) OR ("related to":ti,ab,kw NEAR/3 variable*:ti,ab,kw) OR ("differences were":ti,ab,kw NEAR/3 "explained by":ti,ab,kw) OR ("significant among":ti,ab,kw)) 126125

#13 #3 OR #4 OR #5 OR #6 OR # OR #8 OR #9 OR #10 OR #11 OR #12 234806

#14 #1 AND #2 AND #13 2434

#15 #14 in Cochrane Reviews 105
